# The nucleus of the solitary tract and the coordination of respiratory and sympathetic activities

**DOI:** 10.3389/fphys.2014.00238

**Published:** 2014-06-25

**Authors:** Daniel B. Zoccal, Werner I. Furuya, Mirian Bassi, Débora S. A. Colombari, Eduardo Colombari

**Affiliations:** Department of Physiology and Pathology, School of Dentistry of Araraquara, São Paulo State University (UNESP)Araraquara, Brazil

**Keywords:** NTS, neurotransmission, sympathetic activity, respiration, chemoreflex, cardiorespiratory coupling

## Abstract

It is well known that breathing introduces rhythmical oscillations in the heart rate and arterial pressure levels. Sympathetic oscillations coupled to the respiratory activity have been suggested as an important homeostatic mechanism optimizing tissue perfusion and blood gas uptake/delivery. This respiratory-sympathetic coupling is strengthened in conditions of blood gas challenges (hypoxia and hypercapnia) as a result of the synchronized activation of brainstem respiratory and sympathetic neurons, culminating with the emergence of entrained cardiovascular and respiratory reflex responses. Studies have proposed that the ventrolateral region of the medulla oblongata is a major site of synaptic interaction between respiratory and sympathetic neurons. However, other brainstem regions also play a relevant role in the patterning of respiratory and sympathetic motor outputs. Recent findings suggest that the neurons of the nucleus of the solitary tract (NTS), in the dorsal medulla, are essential for the processing and coordination of respiratory and sympathetic responses to hypoxia. The NTS is the first synaptic station of the cardiorespiratory afferent inputs, including peripheral chemoreceptors, baroreceptors and pulmonary stretch receptors. The synaptic profile of the NTS neurons receiving the excitatory drive from afferent inputs is complex and involves distinct neurotransmitters, including glutamate, ATP and acetylcholine. In the present review we discuss the role of the NTS circuitry in coordinating sympathetic and respiratory reflex responses. We also analyze the neuroplasticity of NTS neurons and their contribution for the development of cardiorespiratory dysfunctions, as observed in neurogenic hypertension, obstructive sleep apnea and metabolic disorders.

## Introduction

A dualistic concept of cardiovascular and respiratory systems still prevails. However, the fundamental organizational precept of cardiorespiratory control may be viewed as a unitary system. Accumulating evidence strongly supports the notion that these systems imperatively operate in a coordinated way to match the processes of pulmonary ventilation and tissue perfusion (Hayano et al., 1996; Giardino et al., 2003; Ben-Tal et al., 2012). Recordings of parasympathetic and sympathetic nerves supplying the heart and blood vessels indicate that the autonomic activity controlling cardiac output and vascular resistance displays robust patterns of discharge entrained with the respiratory cycle (Adrian et al., 1932; Malpas, 1998; Barman and Gebber, 2000; Bouairi et al., 2004; Gilbey, 2007; Grossman and Taylor, 2007). As a consequence, the respiratory-related modulation of cardiovascular parasympathetic and sympathetic activities produces rhythmical oscillations in baseline heart rate (respiratory sinus arrhythmia) and arterial pressure levels (Traube-Hering waves) (Moraes et al., 2012b). The pattern of respiratory modulation of autonomic activity modifies according to the metabolic demand (blood gas changes, for instance) and contributes to generate appropriate respiratory and cardiovascular reflex responses (Dick et al., 2004; Molkov et al., 2011). Therefore, it is undeniable that the respiratory and cardiovascular systems establish interactions to form a unique and dynamic system. In this review, we discuss the potential neuronal sources underpinning the coupling of cardiovascular and respiratory activities, with special attention to the neurons of the nucleus of the solitary tract (NTS), which, due to their anatomical, neurochemical and electrophysiological properties, allow the processing of characteristic, often coupled, patterns of respiratory and autonomic responses to specific physiological and pathological mechanisms.

## Respiratory-sympathetic coupling: ventromedullary neurons and beyond

Over the last decades, efforts have been made to identify the cellular sources and neurochemical mechanisms responsible for the cardiorespiratory coupling. Several studies have focused on the interaction between respiratory and sympathetic activities due to its relevance in the control of arterial pressure and its contribution to the development of arterial hypertension (Zoccal et al., 2008; Simms et al., 2009; Toney et al., 2010). Although the pattern of respiratory-sympathetic coupling may vary according to the animal species, experimental condition (for instance, the presence of anesthesia) and the nerve recorded (Malpas, 1998), baseline sympathetic nerve activity to blood vessels in mammals exhibits phasic bursts that emerge during inspiratory/post-inspiratory phase (Zhou and Gilbey, 1992; Dick et al., 2004; Zoccal et al., 2008). Part of the respiratory oscillations of sympathetic activity is associated with cyclic stimulation of peripheral afferent receptors, including arterial baroreceptors and pulmonary stretch receptors (Bernardi et al., 2001). However, the respiratory rhythmicity of sympathetic activity persists after vagotomy and decerebration (Barman and Gebber, 1980), indicating that the coupling of respiratory and sympathetic activities is generated within the brainstem due to connections between neurons of sympathetic and respiratory systems.

The ventral surface of the medulla oblongata has been pointed out as the main site of synaptic interactions between respiratory and sympathetic neurons. The ventral medulla houses the pre-sympathetic neurons of the rostral ventrolateral medulla (RVLM)—the major source of excitatory inputs to the pre-ganglionic sympathetic neurons of the spinal cord that maintain baseline arterial pressure in adequate levels (Guertzenstein and Silver, 1974; Ross et al., 1984). Intermingled with the RVLM neurons are the respiratory neurons of the ventral respiratory column (VRC), which are considered the kernel of respiratory rhythm and pattern generator (Smith et al., 1991, 2007; Moraes et al., 2011). The VRC is composed by four distinct sub-nuclei: Bötzinger complex (BötC), pre-Bötzinger complex (pre-BötC), rostral ventral respiratory group (rVRG) and caudal ventral respiratory group (cVRG) (Bianchi et al., 1995). Due to their anatomical proximity, it has been suggested that the respiratory neurons of the ventral medulla establish synaptic connections with the pre-sympathetic neurons of the RVLM and generate the respiratory oscillation in the sympathetic activity (McAllen, 1987; Haselton and Guyenet, 1989; Zhou and Gilbey, 1992; Miyawaki et al., 1995; Zoccal et al., 2008).

Recent studies performing intracellular recordings of the RVLM pre-sympathetic neurons using the *in situ* preparation of rats identified different populations of C1 and non-C1 neurons that exhibit spontaneous excitatory or inhibitory post-synaptic potentials phase-locked with the respiratory cycle (Moraes et al., 2013), thus supporting the notion that the RVLM is an important site of respiratory input convergence (Haselton and Guyenet, 1989; Miyawaki et al., 1995). In addition to the RVLM, the GABAergic neurons of the caudal ventrolateral medulla (CVLM), which establish synaptic inputs with the RVLM neurons and provide the tonic inhibitory baroreflex control of sympathetic activity (Schreihofer and Guyenet, 2003), also display distinct firing patterns coupled to the respiratory cycle (Mandel and Schreihofer, 2006). Therefore, there is convincing evidence that the pre-sympathetic neurons of the RVLM, either directly or indirectly through the CVLM, receive the respiratory inputs necessary for the generation of respiratory modulation of sympathetic activity. On the other hand, there are limited data on the literature suggesting the sources of respiratory inputs to the sympathetic neurons of the ventral medulla. Based on the fact the RVLM and CVLM neurons exhibit distinct respiratory-related patterns of discharge (inspiratory peak, post-inspiratory peak and inspiratory depression) (Haselton and Guyenet, 1989; Mandel and Schreihofer, 2006; Moraes et al., 2013), it is possible to speculate that both inspiratory and expiratory neurons of the VRC may establish excitatory and inhibitory synapses with the sympathetic neurons. In agreement with this idea, anatomical studies have evidenced axon varicosities from the BötC neurons closely apposed to the RVLM neurons, strongly suggesting synaptic contacts between these two neuronal populations (Sun et al., 1997). Studies showing that ponto-medullary transection eliminated baseline respiratory-sympathetic coupling also suggest that the respiratory neurons of the pons, which send projections to ventromedullary regions (Dobbins and Feldman, 1994; Kubo et al., 1998), may contribute to baseline respiratory-sympathetic coupling (Baekey et al., 2008).

**Figure 1:**
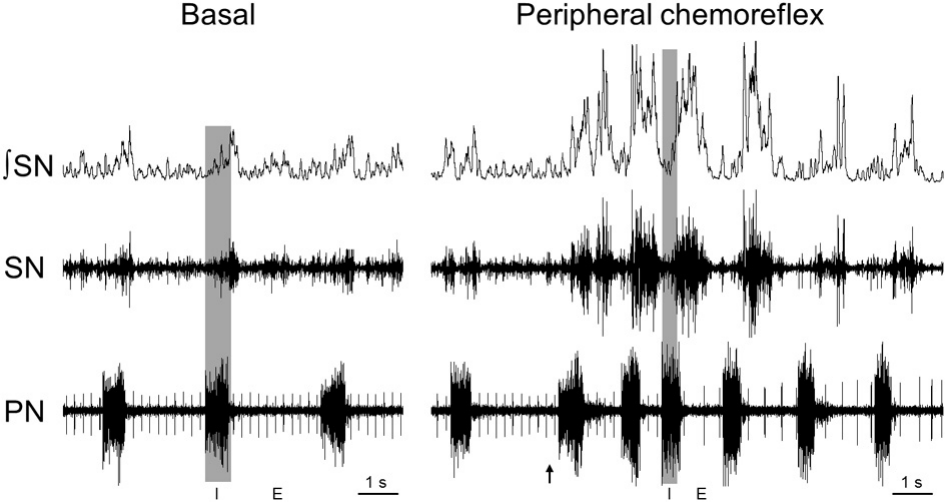
**Respiratory-sympathetic coupling at basal conditions and during peripheral chemoreflex activation**. Raw and integrated (⨛) recordings obtained in a decerebrated, arterially-perfused *in situ* rat preparation (for details, please see Zoccal et al., 2008) showing the coupling of phrenic (PN) and thoracic sympathetic nerve activity (SN) at basal conditions (normocapnia) and during peripheral chemoreflex activation, evoked by intra-arterial administration of potassium cyanide (0.05%, 50 μL; arrow). The shaded gray area delimitates the inspiratory phase (I; coincident with phrenic burst) while the phrenic burst interval correspond to expiratory phase (E).

In addition to the lack of clear anatomical and functional data showing the neural substrates responsible for the generation of respiratory modulation of sympathetic outflow at resting conditions (eupnea), changes in the respiratory pattern modify the pattern of respiratory-sympathetic coupling. For instance, peripheral chemoreceptor activation by cytotoxic (with cyanide) or hypoxic hypoxia reflexly induces marked increases in inspiratory and expiratory motor activities combined with a sympatho-excitatory response (Braga et al., 2007; Moraes et al., 2012a). This increase in sympathetic activity is characterized by the emergence of high amplitude bursts during the post-inspiratory phase (Dick et al., 2004; Mandel and Schreihofer, 2009; Costa-Silva et al., 2010)—a discharge pattern different from that observed at baseline conditions (Figure 1). Changes in the respiratory pattern and in the respiratory-sympathetic coupling are also observed in conditions of hypercapnia, which causes an increase in the sympathetic activity during the late part of expiratory period (prior to the phrenic burst) coupled with the generation of active expiratory pattern (Boczek-Funcke et al., 1992; Molkov et al., 2011). Therefore, multiple synaptic interactions may exist between respiratory and sympathetic neurons, and either the recruitment or the strengthening of these interactions essentially depends on the activation of neurons regulating the respiratory pattern.

Accordingly, in spite of the unquestionable role of the ventro-medullary neurons on the control and integration of respiratory and sympathetic activities, other brain areas must be considered as critical pieces of the circuitry regulating the coupling of respiratory and sympathetic activities. In this scenario, the neurons of the nucleus of the solitary tract (NTS) may play a relevant role for the coordination of the respiratory and sympathetic activities, especially in conditions of metabolic challenges, probably by acting as an integrative center to promote the activation of specific pathways due to the combination of neurochemical and neuroanatomical features.

## The NTS circuitry and its integrative role

The NTS is formed by heterogeneous groups of neurons located in the dorsolateral medulla, extending from the level of caudal portion of the facial nucleus to the caudal portion of the pyramidal decussation (Loewy, 1990; Dampney, 1994). Previous studies have demonstrated that the NTS plays a key role in the processing of visceral afferent information and transmission to other nuclei in the brainstem, forebrain and spinal cord (for review, see Andresen and Kunze, 1994; Sapru, 1996; Johnson and Thunhorst, 1997; Grill and Hayes, 2009). With respect to the cardiovascular and respiratory systems, the majority of the cardiorespiratory afferents converges preferentially to two different regions of the NTS: the intermediate NTS (iNTS), at the level of the area postrema, and the caudal NTS (cNTS), located caudal to the *calamus scriptorius* (Kumada et al., 1990; Loewy, 1990; Van Giersbergen et al., 1992). There is evidence supporting the notion that the iNTS and cNTS 2nd-order neurons are organized in clusters according to the afferent sensory information that they received. For instance, the neurons of the iNTS receive mainly afferent information from the arterial baroreceptors and from the slow-adapting pulmonary stretch receptors (SARs) whilst afferent inputs from peripheral chemoreceptors and rapid-adapting pulmonary stretch receptors (RARs) converge to the neurons of the cNTS (Mifflin et al., 1988; Mifflin, 1992; Machado, 2001; Kubin et al., 2006). Therefore, depending on the sensory information, distinct groups of NTS 2nd-order neurons are activated in order to recruit specific efferent pathways (Aicher et al., 1996; Bailey et al., 2006; Alheid et al., 2011; Song et al., 2011).

In association with the afferent-oriented organization, several bioactive molecules were identified in the NTS region, including amino acids (L-glutamate, GABA, glycine), biogenic amines (noradrenaline, serotonin, acetylcholine), purines (ATP and ADP) and peptides (angiotensin II, vasopressin, oxytocin), with relevant role in the neurotransmission and neuromodulation (for review, see Andresen and Kunze, 1994). These substances may act either post-synaptically or exert their function on pre-synaptic terminals modulating the release of other transmitters (Andresen and Yang, 1990; Shigetomi and Kato, 2004; Peters et al., 2008). Moreover, distinct 2nd-order neurons of the NTS exhibit different electrophysiological properties (De Castro et al., 1994; Doyle et al., 2004; Accorsi-Mendonca et al., 2011) that may contribute to differential timing control of the viscera-sensory information.

The combination of anatomical, neurochemical and electrophysiological features of the NTS neurons allow that this nucleus selectively integrates the sensory information and recruits specific neural pathways to generate appropriate cardiorespiratory responses. Given this significance, it is evident that changes in the normal functioning of the NTS neurons have great impact on the control of sympathetic and respiratory activities with pathological relevance, such as observed in animal models of arterial hypertension (Sato et al., 2002; Zhang and Mifflin, 2010) and in animals exposed to chronic intermittent hypoxia (Reeves et al., 2003; Kline et al., 2007; Almado et al., 2012). In the following sections, we discuss the potential role of the NTS in coordinating sympathetic and respiratory reflex responses, focusing on the neurotransmission and on the efferent brainstem pathways. The involvement of neural pathways from the NTS to mid- and forebrain regions will not be discussed in this review, since their involvement in the respiratory-sympathetic coupling is not clear.

## The NTS and the respiratory-sympathetic coupling during peripheral chemoreflex activation

It is well known that prolonged exposure to low partial pressure of arterial oxygen (PaO_2_) may produce persistent changes in the cellular activity leading to tissue dysfunction and death (Peña and Ramirez, 2005). To prevent this, a reflex system, named the peripheral chemoreflex, was developed in order to evoke appropriated and coordinated cardiovascular and respiratory responses to counteract the fall in PaO_2_. During hypoxia, the reduction of PaO_2_ is detected by chemosensitive cells, mainly located in the carotid bodies (Lahiri et al., 2006), which, in turn, send excitatory inputs to the 2nd-order neurons of the cNTS (Teppema et al., 1997; Cruz et al., 2010). These cNTS neurons are responsible for the integration and transmission of the peripheral chemoreceptor signals to other brain regions, leading to the emergence of sympathetic, parasympathetic and respiratory reflex responses that increase arterial pressure, reduce heart rate and enhance ventilation, respectively (Haibara et al., 1995; Colombari et al., 1996; Sapru, 1996). Previous studies suggested that the sympatho-excitatory response of the peripheral chemoreflex is dependent on the activation of NTS neurons that send excitatory inputs to the pre-sympathetic neurons of the RVLM (Aicher et al., 1996; Koshiya and Guyenet, 1996a). Studies performed in anesthetized rats (Dick et al., 2004; Mandel and Schreihofer, 2006) and unanesthetized *in situ* rat preparations (Costa-Silva et al., 2010) verified that the peripheral chemoreflex-induced sympathetic response is characterized by the emergence of higher amplitude bursts during post-inspiratory phase followed by inhibition during early inspiration (Figure 1). This pattern of sympathetic reflex response occurs entrained with the responses of augmented phrenic burst frequency (increased inspiratory drive) and increased abdominal burst amplitude (active expiration) (Moraes et al., 2012a). Therefore, it has been suggested that the sympathetic response to peripheral chemoreflex activation exhibit two components: one independent and another dependent on the activation of respiratory neurons (Koshiya and Guyenet, 1996b; Costa-Silva et al., 2010). Recent evidence suggests that the processing and coupling of the sympathetic and respiratory responses of peripheral chemoreflex rely on the recruitment of different neuronal population and/or neurochemical mechanisms in the cNTS (Braga et al., 2007; Furuya et al., 2014).

Previous studies performed in anesthetized animals proposed that L-glutamate is a major excitatory neurotransmitter released by the peripheral chemoreceptor afferents in the cNTS (Zhang and Mifflin, 1993; Sapru, 1996; Gozal et al., 1999). However, studies performed in unanesthetized rats and in the *in situ* rat preparation failed to block the sympatho-excitatory and respiratory responses elicited by peripheral chemoreceptor activation after the antagonism of both ionotropic and metabotropic glutamatergic receptors (Machado and Bonagamba, 2005; Braga and Machado, 2006), indicating that neurontransmitters other than L-glutamate mediate the processing of these responses in the cNTS. Afterwards, it was verified that the antagonism of both ionotropic glutamate and P2-purinergic receptors in the cNTS reduced significantly the magnitude of sympathetic response to peripheral chemoreflex activation without affecting the tachypneic response (Braga et al., 2007; Braccialli et al., 2008). Therefore, the processing of sympathetic chemoreflex response by the cNTS neurons involves a complex interaction between L-glutamate and ATP (Accorsi-Mendonca et al., 2009). On the other hand, these neurotransmitters, at the cNTS level, apparently are not essential for the tachypneic reflex response, suggesting the involvement of an additional neurotransmitter system.

Since a functional cholinergic system has been described in the NTS (Ruggiero et al., 1990; Shihara et al., 1999), our group recently assessed the contribution of acetylcholine (ACh) in the processing of peripheral chemoreflex inputs in the cNTS using the arterially-perfused *in situ* rat preparation. We verified that microinjections of ACh in the cNTS did not change mean levels of sympathetic activity but increased phrenic burst frequency (Furuya et al., 2014). In spite of the lack of effects on mean levels of sympathetic activity, microinjections of ACh in the cNTS altered its coupling pattern with the respiratory activity, producing a shift of respiratory-related peak from the inspiratory to the post-inspiratory phase associated with an inhibition during the inspiratory phase (Furuya et al., 2014)—a pattern similar to that evoked by peripheral chemoreflex activation (Dick et al., 2004; Costa-Silva et al., 2010). Moreover, the antagonism of nicotinic receptors in the cNTS prevented the ACh-induced tachypnea and significantly reduced the tachypneic response following chemoreflex activation, without affecting the magnitude of sympatho-excitatory response (Furuya et al., 2014). In addition, the nicotinic antagonism in the cNTS also prevented the ACh-induced change in sympatho-respiratory coupling (Furuya et al., 2014). These data suggest that cholinergic mechanisms of the cNTS, at least in part, contribute to the processing of inspiratory response of the peripheral chemoreflex activation and its entrainment with the sympathetic activity. Recent studies have reported efferent projections from the cNTS to respiratory nuclei of the ventrolateral medulla and dorsolateral pons (Takakura et al., 2006; Alheid et al., 2011; Song et al., 2011) that may be involved with the emergence of respiratory responses of peripheral chemoreflex. However, further studies are still required to elucidate the respiratory nuclei targeted by the cNTS neurons activated by ACh.

**Figure 2:**
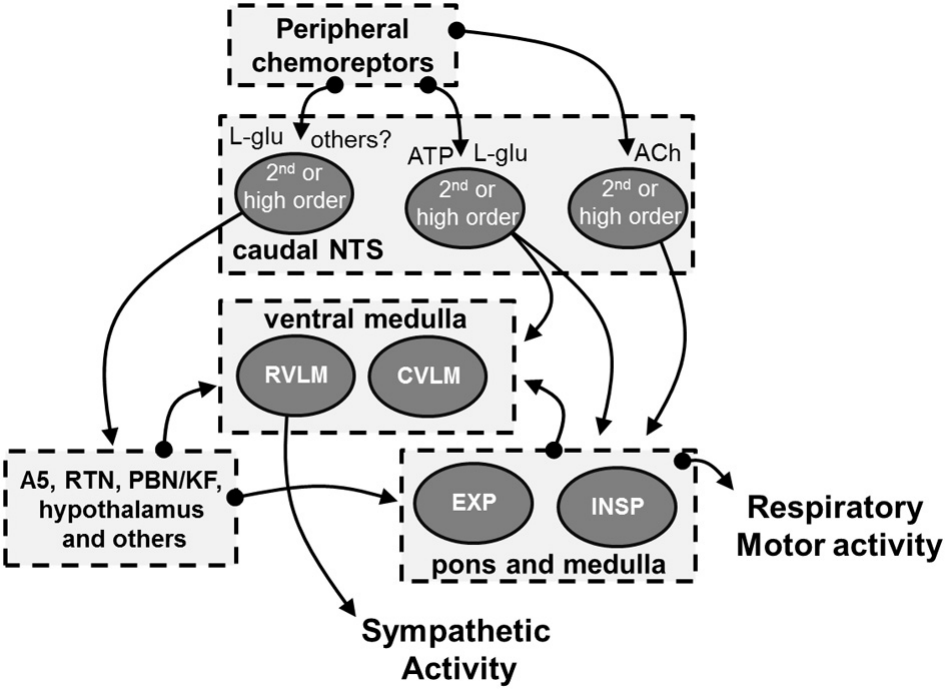
**Schematic drawing showing the possible cellular and neurochemical mechanisms of the cNTS mediating the processing of peripheral chemoreceptors inputs**. The stimulation of peripheral chemoreceptors evokes the release of L-glutamate in the cNTS (Mifflin, 1992; Andresen and Kunze, 1994). Experimental evidence indicates the L-glutamate, in association with ATP, is essential for the processing of sympatho-excitatory response of the peripheral chemoreflex in the cNTS (Machado and Bonagamba, 2005; Braga et al., 2007). It is suggested that both glutamatergic and purinergic systems interact and activate cNTS neurons that send projections to pre-sympathetic neurons of the RVLM (Accorsi-Mendonca et al., 2009, 2013). L-glutamate is also proposed to mediate the activation of other neural pathways that are important for the sympatho-excitatory component of peripheral chemoreflex, including those to the A5 region, retrotrapezoid nucleus (RTN), parabrachial nucleus/Kölliker-Fuse complex (PBN/KF) and hypothalamus (Koshiya and Guyenet, 1994; Olivan et al., 2001; Haibara et al., 2002; Reddy et al., 2005; Takakura et al., 2006; Queiroz et al., 2011; Song et al., 2011; Taxini et al., 2011; King et al., 2012). In addition to L-glutamate and ATP, our recent studies suggest that ACh in the cNTS significantly contributes to the tachypnea and the patterning of sympathetic response of peripheral chemoreflex (Furuya et al., 2014). We hypothesize that ACh activates cNTS neurons that send projections to respiratory neurons of the ventral medulla that, in turn, promotes the stimulation of inspiratory motor activity and the patterning of sympathetic activity. The latter may involve the modulation of the neurons of RVLM and of the caudal ventrolateral medulla (CVLM) (Mandel and Schreihofer, 2009; Moraes et al., 2012c). However, it remains to be elucidated the sources of ACh in the cNTS as well as the efferent pathways activated by ACh in response to peripheral chemoreflex activation.

Accordingly, the cNTS possesses an intrinsic neuronal circuitry that is essential for the precise coordination of evoked sympathetic and respiratory responses during hypoxia (Figure 2). Our recent data (Furuya et al., 2014) combined with previous studies (Braga et al., 2007), suggest that the respiratory and sympathetic components of peripheral chemoreflex, in unanesthetized conditions, are processed by 2nd-order cNTS neurons activated by different neurotransmitters, including L-glutamate, ATP and ACh. Whether ATP and ACh are released by afferent terminals, likewise L-glutamate, or by other cells of the cNTS (glia or neurons, respectively ATP and ACh) (Accorsi-Mendonca et al., 2013) still remain to be elucidated. Nevertheless, the selective activation of the 2nd-order neurons may be essential for the recruitment of specific neural pathways promoting the increase in sympathetic activity mainly during the post-inspiratory phase entrained with the increases in inspiratory and expiratory motor activities. The physiological meaning of the respiratory-sympathetic coupling observed during peripheral chemoreflex activation is not clear and requires further studies to be fully elucidated. Nonetheless, the sympathetic pattern during hypoxia is different from that observed at baseline conditions or during hypercapnia (Molkov et al., 2011), suggesting, therefore, that it may play a relevant role in matching oxygen uptake/delivery and tissue perfusion in conditions of low PaO_2_.

## The NTS and the processing of cardiovascular and pulmonary feedback information

The iNTS neurons receive mainly the afferent information from mechanoreceptors located in the arterial blood vessels and pulmonary walls (Mifflin and Felder, 1990; Kubin et al., 2006). The arterial mechanoreceptors, named as arterial baroreceptors, are located in the aortic arc and carotid artery bifurcation and provide a powerful inhibitory feedback control of sympathetic activity, lowering the sympathetic vascular tonus in conditions of increased arterial pressure (Chan and Sawchenko, 1998; Gordon and Sved, 2002). This sympatho-inhibitory response is mediated by the activation of excitatory 2nd-order neurons of the NTS that, in turn, promotes the inhibition of RVLM pre-sympathetic neurons through the activation of GABAergic neurons of the CVLM (Aicher et al., 1995; Schreihofer and Guyenet, 2003). Several pharmacological and electrophysiological studies suggest that the L-glutamate is the main neurotransmitter released by baroreceptor afferents (Reis et al., 1981; Bailey et al., 2006). However, Machado et al. (2000), reported that the antagonism of ionotropic glutamatergic receptors in the iNTS of awake rats was unable to block totally the hypotensive response to baroreflex activation (induced by electrical stimulation of the aortic depressor nerve), suggesting that the processing of baroreflex afferent information may either involve other regions of the NTS or other neurotransmitter systems than L-glutamate.

In association with the sympatho-inhibitory response, activation of baroreceptors also prolongs expiratory time, reducing the respiratory frequency (Richter and Seller, 1975; Lindsey et al., 1998; Baekey et al., 2010). According to Baekey et al. (2010), the expiratory lengthening induced by arterial baroreceptor activation results from the activation of post-inspiratory neurons of the BötC, which, in turn, inhibit the central inspiratory activity (Smith et al., 2007). Since these post-inspiratory neurons may also establish synaptic contacts with the pre-sympathetic neurons of the RVLM (Sun et al., 1997), the baroreflex-induced activation of inhibitory post-inspiratory neurons of the BötC may additionally contribute to depress the RVLM neuronal activity (Baekey et al., 2010). Besides, with the post-inspiratory activation and the increase in expiratory time, the emergence of inspiratory-related bursts of sympathetic activity is delayed and the sympathetic activity is maintained at low levels. Therefore, in addition to the classical NTS-CVLM-RVLM baroreflex arc, a parallel respiratory-dependent pathway from the NTS to the VRC also contributes to reduce sympathetic activity in conditions of high arterial pressure. It remains to be elucidated whether this respiratory baroreflex pathway relies on the different subset of 2nd-order neurons (for instance, neurons receiving SAR afferent information, as described below) or recruits neurotransmitter other than L-glutamate (Machado et al., 2000; Baekey et al., 2010).

Mechanoreceptors located mainly in pulmonary walls, including the SARs and RARs, are important to reflexly control the central respiratory command according to lung volume (for further review, see Kubin et al., 2006). Although both receptors are sensitive to pulmonary wall distension, the lung inflation feedback control is evoked mainly by SAR afferent inputs (Bonham et al., 1993). The vagal afferents from SARs terminates predominantly in the ventrolateral portion of the iNTS (Kalia and Mesulam, 1980). In rats, different populations of neurons are suggested to receive the afferent information from SARs: (i) inspiratory neurons that fire during central inspiratory drive and are sensitive to vagal stimulation (either excited or inhibited); (ii) pump cells (P cells) that present action potentials in phase with lung inflation cycle, but not with central inspiratory rhythm, and are activated when lungs are overinflated (Bonham and McCrimmon, 1990; De Castro et al., 1994). SARs stimulation initiates the Hering-Breuer reflex and promotes the suppression of inspiratory motor activity and prolongs the expiratory phase (Breuer, 1868; Hering, 1868; Backman et al., 1984; Kubin et al., 2006). The removal of pulmonary feedback control, through vagotomy, results in large increases of inspiratory amplitude and duration (Kubin et al., 2006), evidencing an important role of the SARs feedback information for baseline control of respiratory phase transition and duration.

P cells of the iNTS are thought to be the main cellular source integrating and transmitting the SAR afferent information to pontine and medullary respiratory neurons (McCrimmon et al., 1987; Bonham et al., 1993; Ezure et al., 2002; Molkov et al., 2013), leading to the activation of the post-inspiratory neurons of the BötC that, in turn, inhibit the inspiratory neuronal activity (Hayashi et al., 1996). By the fact that the antagonism of ionotropic glutamatergic receptors in the iNTS of anesthetized animals greatly attenuates baseline activity of P cells and impairs the Hering-Breuer respiratory reflex responses (Bonham and McCrimmon, 1990; Bonham et al., 1993), it has been suggested that L-glutamate is the major neurotransmitter release by SAR vagal afferent in the iNTS. Microinjections of glutamatergic agonists into the iNTS excite P cells and promote apnea and expiratory lengthening like Hering-Breuer reflex (Bonham et al., 1993; Gourine et al., 2008), corroborating the idea that L-glutamate is released by SAR afferent inputs in the iNTS. In addition to L-glutamate, there is evidence that other neurotransmitters may play a role in the processing of pulmonary afferent information at the level of iNTS. Studies by Gourine et al. (2008) demonstrated that the concentrations of ATP and L-glutamate in the iNTS increase in phase to lung inflation and independently on central respiratory drive, suggesting that SAR afferents may release both neurotransmitters. Also, injections of ATP in the iNTS mimic the respiratory responses induced by lung inflation (Antunes et al., 2005; Gourine et al., 2008) and the antagonism of purinergic receptors significantly reduced basal activity of P cells (Gourine et al., 2008). These results suggest that an interaction between glutamatergic and purinergic systems may mediate the processing of pulmonary feedback information at the level of iNTS.

Lung inflation also elicits a decrease in sympathetic activity and vasodilation in addition to the respiratory responses (Gerber and Polosa, 1978; Sellden et al., 1987; Yu et al., 1990), suggesting a possible correlation between the respiratory and sympathetic responses to SAR activation. Since L-glutamate is suggested to be the major neurotransmitter release by SAR afferents (Bonham et al., 1993), it is possible to speculate that glutamatergic activation of P cells also mediates the reduction of sympathetic activity associated with expiratory prolongation. In agreement with that, studies have demonstrated that microinjections of L-glutamate in the iNTS produce coupled inspiratory inhibition, expiratory lengthening and sympatho-inhibition (Berger et al., 1995; Marchenko and Sapru, 2000). Alternatively, it is possible that the sympathetic and inspiratory inhibitory responses to lung inflation rely on interactions with neurons that are part of the baroreflex pathway, which are also located in the iNTS and are activated by L-glutamate (Bailey et al., 2006). Both possibilities, although not proven experimentally, indicate that the iNTS possesses a complex neuronal circuitry that is important to coupled respiratory and sympathetic responses to cardiorespiratory mechanoreceptor activation.

We recently verified that microinjections of ACh in the iNTS reduce phrenic burst frequency, due to an increase in expiratory time, associated with reductions in sympathetic activity (Furuya et al., 2014). Interestingly, this cholinergic system of the iNTS apparently is not involved in the processing of the baroreflex responses because the antagonism of cholinergic receptors in the iNTS did not prevent the sympathetic and respiratory responses elicited by increase in pressure (Furuya et al., 2014). These findings suggest that the cholinergic neurotransmission in the iNTS may also contribute to the processing of respiratory and sympathetic responses of Hering-Breuer reflex. Nonetheless, this possibility still requires further experiments to be proven.

Therefore, the respiratory and sympathetic changes induced by Hering-Breuer activation may involve the release/co-release of distinct neurotransmitters, which may interact and activate either excitatory or inhibitory P cells (Kubin et al., 2006) or different neuronal populations (P cells and baroreflex sensitive neurons) within the iNTS.

## NTS neuroplasticity and the development of cardiorespiratory dysfunctions

In response to intensive stimuli, the afferents and neurons of the NTS may exhibit plastic changes (Zhou et al., 1997; Chen et al., 1999; Kline, 2008) that, in turn, modify the control of respiratory and sympathetic activities. Depending on the degree of plasticity, the changes in the NTS neuronal activity may persist and contribute to the development and/or maintenance of cardiorespiratory dysfunctions. This may be the case of pathological conditions associated with potentiation of peripheral chemoreflex responses and sympathetic overactivity (Narkiewicz et al., 1998; Niewinski et al., 2013). Therefore, the understanding of the mechanisms promoting plastic changes in the NTS circuitry associated with pathological conditions may help to develop potential therapeutic venues to treat cardiorespiratory diseases, as discussed below.

### Neurogenic hypertension

There is compelling evidence indicating that hyperactivity of the sympathetic nervous system is an important mechanism producing the chronic elevation of arterial pressure in hypertensive patients (Staessen et al., 2003), especially those that are resistant to concurrent anti-hypertensive treatment (Esler, 2012). Recent studies indicate that the surgical removal of the carotid body peripheral chemoreceptors of juvenile (4-week old) spontaneous hypertensive rats (SHR), an experimental model for neurogenic hypertension, attenuates the development of hypertension (Abdala et al., 2012). In adult SHR rats (12-week old), the carotid body denervation significantly reduces arterial pressure and renal sympathetic nerve activity levels (McBryde et al., 2013). The high levels of sympathetic activity in SHRs exhibit an amplified respiratory-sympathetic coupling, with augmented sympathetic activity mainly during the inspiratory phase (Czyzyk-Krzeska and Trzebski, 1990; Simms et al., 2009) that is also reduced with the removal of carotid body chemoreceptors (McBryde et al., 2013). Based on that, it has been proposed that heightened peripheral chemoreceptor activity contributes to elevate baseline levels of sympathetic activity and magnify the respiratory modulation of sympathetic activity in SHR rats. Studies by Sato et al. (2001) have shown that lesions of the cNTS also produce a significant fall in the arterial pressure level in adult SHR, but not in normotensive rats, suggesting that the development of hypertension in this model may involve neuroplasticity within the NTS. In agreement with this hypothesis, there are several studies demonstrating relevant changes in the mechanisms of neurotransmission and neuromodulation in the NTS of SHR rats, including glutamate (Aicher et al., 2003), angiontesin II (Shan et al., 2013), GABA (Mei et al., 2003; Spary et al., 2008), nitric oxide (Hirooka et al., 2003) and inflammatory molecules (Waki et al., 2008). Altogether, these findings indicate that neurogenic hypertension is causally associated with potentiation of peripheral chemoreflex, in which plastic changes of cNTS neurons receiving the afferent inputs from the carotid bodies importantly contribute to elevate baseline sympathetic activity and strength respiratory-sympathetic coupling. The contribution of amplified respiratory-sympathetic coupling for the development and maintenance of arterial hypertension in SHR rats is still under investigation and is matter of debate (Fatouleh and Macefield, 2011; Moraes et al., 2014).

### Chronic intermittent hypoxia

Sensitization of the peripheral chemoreflex is observed in patients suffering obstructive sleep apnea (OSA). Due to recurrent collapses of upper airways, OSA patients frequently experience intermittent episodes of hypoxia during sleep (Dempsey et al., 2010). Overtime, untreated OSA patients develop arterial hypertension associated with high levels of sympathetic activity (Somers, 1995) and tonic chemoreflex activation (Narkiewicz et al., 1998). In rodent models, the exposure to chronic intermittent hypoxia (CIH) causes a sustained increase in baseline arterial pressure that is prevented by carotid body denervation (Fletcher et al., 1992). The hypertension induced by CIH in rats is associated with high levels of baseline sympathetic activity (Zoccal et al., 2007, 2008, 2009), enhanced baseline frequency of discharge of RVLM pre-sympathetic neurons (Moraes et al., 2013), augmented peripheral chemoreflex responses (Peng et al., 2003; Rey et al., 2004; Braga et al., 2006), changes in the respiratory pattern and strengthened respiratory-sympathetic coupling (Zoccal et al., 2008). Therefore, intermittent activation of the peripheral chemoreceptors, as observed in OSA patients, introduces persistent changes in the neural pathways of peripheral chemoreflex that culminate with the development of cardiorespiratory dysfunctions.

With respect to the NTS, studies *in vitro* evidenced that AMPA-evoked currents of isolated 2nd-order neurons of the cNTS are enhanced in rats exposed to CIH (De Paula et al., 2007). Our previous studies demonstrated that CIH rats exhibit augmented sympatho-excitatory and blunted phrenic apneic responses to microinjections of L-glutamate in the cNTS in association with increased densities of NMDA and non-NMDA receptor subunities within the cNTS (Costa-Silva et al., 2012). Altogether, these studies indicate that CIH exposure promotes functional changes in the glutamatergic neurontrasmission in the cNTS. In addition to the glutamatergic mechanisms, Zhang et al. (2008) reported that the outward currents mediated by ATP-sensitive potassium channels (K_ATP_) of 2nd-order neurons of the peripheral chemoreceptors of CIH rats are reduced—a fact that may increase the excitability of these neurons. Accordingly, CIH exposure appears to alter post-synaptic mechanisms in neurons of the cNTS, amplifying the excitatory responses to glutamatergic receptor activation.

Kline et al. (2007) have also documented that neurons of the cNTS of rats submitted to CIH exhibit a higher basal frequency of discharge due to an augmented spontaneous neurotransmitter release by pre-synaptic terminals. Therefore, increased spontaneous neurotransmitter release combined with amplified post-synaptic glutamatergic excitatory responses may represent an important mechanism producing enhanced basal and evoked activity of cNTS neurons after CIH. As a consequence, higher excitatory drive is transmitted to downstream sympathetic and respiratory chemoreflex pathways, which contribute, at least in part, to the development of cardiorespiratory dysfunctions associated with CIH exposure. So far, there are no studies elucidating the involvement of other neurotransmitters or neuromodulators of the cNTS (for instance ATP or ACh) in the context of CIH. This represents important possibilities to better understand the contribution of the cNTS mechanisms to elevate baseline sympathetic activity or modify the respiratory pattern and the respiratory-sympathetic coupling in CIH rats, since the overactivity of RVLM pre-sympathetic neurons induced by CIH mainly depends on synaptic inputs rather than changes in their intrinsic properties (Moraes et al., 2013).

### Obesity

Additionally to the classical neurochemical mechanisms, there is evidence that peripheral molecules interfere with the activity of the NTS neurons and then modify sympathetic and respiratory activities. This peripheral-central crosstalk appears to have significance in the context of the cardiovascular dysfunctions related to obesity. Obese individuals may develop arterial hypertension associated with high levels of sympathetic activity (Hall et al., 2010), by mechanisms not completely understood. Adipocytes are considered as an endocrine tissue producing several substances including interleukin-6, tumor necrosis factors-α, adiponectin and leptin (Kershaw and Flier, 2004; Galic et al., 2010). Attention has been given to the effects of leptin on the central mechanisms controlling autonomic and respiratory activities. In addition to its central effects suppressing appetite (Grill, 2006), it was shown that leptin exerts excitatory effects on central nuclei controlling sympathetic (Rahmouni et al., 2005; Mark et al., 2009) and respiratory activities (Inyushkina et al., 2010; Malli et al., 2010; Bassi et al., 2012, 2014; Chang et al., 2013), increasing baseline sympathetic activity and stimulating breathing. By the fact that leptin levels are enhanced in obese subjects (Considine et al., 1996), this hormone can be considered as a relevant candidate acting on central nervous system and contributing to the cardiovascular changes associated with obesity.

Systemic administration of leptin has been shown to increase *c-fos* expression in neurons of the cNTS (Elmquist et al., 1998; Elias et al., 2000) and enhance phrenic burst amplitude (Chang et al., 2013). Morevoer, microinjections of leptin in the NTS increase renal sympathetic activity (Mark et al., 2009) and ventilation (Inyushkin et al., 2009). Therefore, the NTS is a potential target for action of circulating leptin. Importantly, both obesity and high leptin levels have been observed in OSA patients, who present a high risk to develop arterial hypertension than non-obese OSA patients (Schafer et al., 2002; Harsch et al., 2003; Patel et al., 2004). Therefore, a positive interaction between intermittent hypoxia, peripheral chemoreflex activation and leptin effects may be considered. In this regard, it was reported that the sympatho-excitatory responses induced by either activation of peripheral chemoreceptors or microinjections of L-glutamate in the cNTS are potentiated by previous microinjections of leptin in the cNTS (Ciriello and Moreau, 2012). Thus leptin enhances the sympatho-excitatory responses to peripheral chemoreflex activation through its actions on cNTS neurons involved with the processing of peripheral chemoreceptor inputs. The effects of leptin on the chemoreflex-induced respiratory response and on respiratory-sympathetic coupling are still to be determined.

## Concluding remarks

Although the precise physiological meaning of the respiratory-sympathetic coupling in different conditions (resting, metabolic challenges and physical exercise) is still under debate (Hayano et al., 1996; Ben-Tal et al., 2012), there is evidence showing that modifications in the central mechanisms underlying the interactions between respiratory and sympathetic neurons are associated with the development of sympathetic overactivity (Zoccal et al., 2008; Simms et al., 2009; Toney et al., 2010). In this regard, the identification of neuronal sources and targets as well as the corresponding neurochemical mechanisms underlying the respiratory-sympathetic coupling is required for a better comprehension of this phenomenon and its pathological implications. In addition to ventral medullary neurons, the NTS neurons play an essential role in coordinating respiratory and sympathetic adjustments in response to activation of peripheral cardiovascular and pulmonary afferent inputs, such as the baroreceptors, the pulmonary stretch receptors and especially the peripheral chemoreceptors. Due to its afferent-based organization, neurons of specific sub-regions of the NTS may respond to different stimuli and generate specific patterns of responses due to the recruitment of specific downstream sympathetic and respiratory pathways.

## Author contributions

Daniel B. Zoccal designed the manuscript. Daniel B. Zoccal, Werner I. Furuya, Mirian Bassi, Débora S. A. Colombari and Eduardo Colombari drafted and revised the manuscript. Daniel B. Zoccal prepared the figures. All authors approved the final version of the manuscript.

### Conflict of interest statement

The authors declare that the research was conducted in the absence of any commercial or financial relationships that could be construed as a potential conflict of interest.

## References

[B1] Abdala A. P. McBryde F. D. Marina N. Hendy E. B. Engelman Z. J. Fudim M. (2012). Hypertension is critically dependent on the carotid body input in the spontaneously hypertensive rat. J. Physiol. 590, 4269–4277 10.1113/jphysiol.2012.237800 22687617 PMC3473284

[B2] Accorsi-Mendonca D. Bonagamba L. G. Leao R. M. Machado B. H. (2009). Are L-glutamate and ATP cotransmitters of the peripheral chemoreflex in the rat nucleus tractus solitarius? Exp. Physiol. 94, 38–45 10.1113/expphysiol.2008.043653 18931046

[B3] Accorsi-Mendonca D. Castania J. A. Bonagamba L. G. Machado B. H. Leao R. M. (2011). Synaptic profile of nucleus tractus solitarius neurons involved with the peripheral chemoreflex pathways. Neuroscience 197, 107–120 10.1016/j.neuroscience.2011.08.054 21963868

[B4] Accorsi-Mendonca D. Zoccal D. B. Bonagamba L. G. Machado B. H. (2013). Glial cells modulate the synaptic transmission of NTS neurons sending projections to ventral medulla of Wistar rats. Physiol. Rep. 1:e00080 10.1002/phy2.80 24303152 PMC3831896

[B5] Adrian E. D. Bronk D. W. Phillips G. (1932). Discharges in mammalian sympathetic nerves. J. Physiol. 74, 115–133 16994262 10.1113/jphysiol.1932.sp002832 PMC1394445

[B6] Aicher S. A. Kurucz O. S. Reis D. J. Milner T. A. (1995). Nucleus tractus solitarius efferent terminals synapse on neurons in the caudal ventrolateral medulla that project to the rostral ventrolateral medulla. Brain Res. 693, 51–63 10.1016/0006-8993(95)00660-I 8653421

[B7] Aicher S. A. Saravay R. H. Cravo S. Jeske I. Morrison S. F. Reis D. J. (1996). Monosynaptic projections from the nucleus tractus solitarii to C1 adrenergic neurons in the rostral ventrolateral medulla: comparison with input from the caudal ventrolateral medulla. J. Comp. Neurol. 373, 62–75 10.1002/(SICI)1096-9861(19960909)373:1<62::AID-CNE6>3.0.CO;2-B 8876463

[B8] Aicher S. A. Sharma S. Mitchell J. L. (2003). Structural changes in AMPA-receptive neurons in the nucleus of the solitary tract of spontaneously hypertensive rats. Hypertension 41, 1246–1252 10.1161/01.HYP.0000069007.98987.E0 12695422

[B9] Alheid G. F. Jiao W. McCrimmon D. R. (2011). Caudal nuclei of the rat nucleus of the solitary tract differentially innervate respiratory compartments within the ventrolateral medulla. Neuroscience 190, 207–227 10.1016/j.neuroscience.2011.06.005 21704133 PMC3169098

[B10] Almado C. E. Machado B. H. Leao R. M. (2012). Chronic intermittent hypoxia depresses afferent neurotransmission in NTS neurons by a reduction in the number of active synapses. J. Neurosci. 32, 16736–16746 10.1523/JNEUROSCI.2654-12.2012 23175827 PMC6621780

[B11] Andresen M. C. Kunze D. L. (1994). Nucleus tractus solitarius—gateway to neural circulatory control. Annu. Rev. Physiol. 56, 93–116 10.1146/annurev.ph.56.030194.000521 7912060

[B12] Andresen M. C. Yang M. Y. (1990). Non-NMDA receptors mediate sensory afferent synaptic transmission in medial nucleus tractus solitarius. Am. J. Physiol. 259, H1307–H1311 1977326 10.1152/ajpheart.1990.259.4.H1307

[B13] Antunes V. R. Bonagamba L. G. Machado B. H. (2005). Hemodynamic and respiratory responses to microinjection of ATP into the intermediate and caudal NTS of awake rats. Brain Res. 1032, 85–93 10.1016/j.brainres.2004.10.048 15680945

[B14] Backman S. B. Anders C. Ballantyne D. Rohrig N. Camerer H. Mifflin S. (1984). Evidence for a monosynaptic connection between slowly adapting pulmonary stretch receptor afferents and inspiratory beta neurones. Pflugers Arch. 402, 129–136 10.1007/BF00583324 6527937

[B15] Baekey D. M. Dick T. E. Paton J. F. (2008). Pontomedullary transection attenuates central respiratory modulation of sympathetic discharge, heart rate and the baroreceptor reflex in the *in situ* rat preparation. Exp. Physiol. 93, 803–816 10.1113/expphysiol.2007.041400 18344259

[B16] Baekey D. M. Molkov Y. I. Paton J. F. Rybak I. A. Dick T. E. (2010). Effect of baroreceptor stimulation on the respiratory pattern: insights into respiratory-sympathetic interactions. Respir. Physiol. Neurobiol. 174, 135–145 10.1016/j.resp.2010.09.006 20837166 PMC3691868

[B17] Bailey T. W. Hermes S. M. Andresen M. C. Aicher S. A. (2006). Cranial visceral afferent pathways through the nucleus of the solitary tract to caudal ventrolateral medulla or paraventricular hypothalamus: target-specific synaptic reliability and convergence patterns. J. Neurosci. 26, 11893–11902 10.1523/JNEUROSCI.2044-06.2006 17108163 PMC6674856

[B18] Barman S. M. Gebber G. L. (1980). Sympathetic nerve rhythm of brain stem origin. Am. J. Physiol. 239, R42–R47 6249131 10.1152/ajpregu.1980.239.1.R42

[B19] Barman S. M. Gebber G. L. (2000). “Rapid” rhythmic discharges of sympathetic nerves: sources, mechanisms of generation, and physiological Relevance. J. Biol. Rhythms 15, 365–379 10.1177/074873000129001468 11039915

[B20] Bassi M. Furuya W. I. Menani J. V. Colombari D. S. Do Carmo J. M. Da Silva A. A. (2014). Leptin into the ventrolateral medulla facilitates chemorespiratory response in leptin-deficient (ob/ob) mice. Acta Physiol. (Oxf). 211, 240–248 10.1111/apha.12257 24521430 PMC4365783

[B21] Bassi M. Giusti H. Leite C. M. Anselmo-Franci J. A. Do Carmo J. M. Da Silva A. A. (2012). Central leptin replacement enhances chemorespiratory responses in leptin-deficient mice independent of changes in body weight. Pflugers Arch. 464, 145–153 10.1007/s00424-012-1111-1 22585210 PMC4077668

[B22] Ben-Tal A. Shamailov S. S. Paton J. F. (2012). Evaluating the physiological significance of respiratory sinus arrhythmia: looking beyond ventilation-perfusion efficiency. J. Physiol. 590, 1989–2008 10.1113/jphysiol.2011.222422 22289913 PMC3573317

[B23] Berger I. Gillis R. A. Vitagliano S. Panico W. H. Magee S. Kelly M. (1995). NMDA receptors are involved at the ventrolateral nucleus tractus solitarii for termination of inspiration. Eur. J. Pharmacol. 277, 195–208 10.1016/0014-2999(95)00073-T 7493609

[B24] Bernardi L. Porta C. Gabutti A. Spicuzza L. Sleight P. (2001). Modulatory effects of respiration. Auton. Neurosci. 90, 47–56 10.1016/S1566-0702(01)00267-3 11485292

[B25] Bianchi A. L. Denavit-Saubie M. Champagnat J. (1995). Central control of breathing in mammals: neuronal circuitry, membrane properties, and neurotransmitters. Physiol. Rev. 75, 1–45 7831394 10.1152/physrev.1995.75.1.1

[B26] Boczek-Funcke A. Dembowsky K. Habler H. J. Janig W. Michaelis M. (1992). Respiratory-related activity patterns in preganglionic neurones projecting into the cat cervical sympathetic trunk. J. Physiol. 457, 277–296 1297836 10.1113/jphysiol.1992.sp019378 PMC1175731

[B27] Bonham A. C. Coles S. K. McCrimmon D. R. (1993). Pulmonary stretch receptor afferents activate excitatory amino acid receptors in the nucleus tractus solitarii in rats. J. Physiol. 464, 725–745 8229827 10.1113/jphysiol.1993.sp019660 PMC1175411

[B28] Bonham A. C. McCrimmon D. R. (1990). Neurones in a discrete region of the nucleus tractus solitarius are required for the Breuer-Hering reflex in rat. J. Physiol. 427, 261–280 2213599 10.1113/jphysiol.1990.sp018171 PMC1189930

[B29] Bouairi E. Neff R. Evans C. Gold A. Andresen M. C. Mendelowitz D. (2004). Respiratory sinus arrhythmia in freely moving and anesthetized rats. J. Appl. Physiol. 97, 1431–1436 10.1152/japplphysiol.00277.2004 15155710

[B30] Braccialli A. L. Bonagamba L. G. Machado B. H. (2008). Glutamatergic and purinergic mechanisms on respiratory modulation in the caudal NTS of awake rats. Respir. Physiol. Neurobiol. 161, 246–252 10.1016/j.resp.2008.02.011 18395497

[B31] Braga V. A. Machado B. H. (2006). Chemoreflex sympathoexcitation was not altered by the antagonism of glutamate receptors in the commissural nucleus tractus solitarii in the working heart-brainstem preparation of rats. Exp. Physiol. 91, 551–559 10.1113/expphysiol.2005.033100 16452122

[B32] Braga V. A. Soriano R. N. Braccialli A. L. De Paula P. M. Bonagamba L. G. Paton J. F. (2007). Involvement of L-glutamate and ATP in the neurotransmission of the sympathoexcitatory component of the chemoreflex in the commissural nucleus tractus solitarii of awake rats and in the working heart-brainstem preparation. J. Physiol. 581, 1129–1145 10.1113/jphysiol.2007.129031 17395636 PMC2170832

[B33] Braga V. A. Soriano R. N. Machado B. H. (2006). Sympathoexcitatory response to peripheral chemoreflex activation is enhanced in juvenile rats exposed to chronic intermittent hypoxia. Exp. Physiol. 91, 1025–1031 10.1113/expphysiol.2006.034868 16959820

[B34] Breuer J. (1868). Die selbststeurung der athmung durch nervus vagus. Akad. Wiss. Wien (II) 58, 909–937

[B35] Chan R. K. Sawchenko P. E. (1998). Organization and transmitter specificity of medullary neurons activated by sustained hypertension: implications for understanding baroreceptor reflex circuitry. J. Neurosci. 18, 371–387 9412514 10.1523/JNEUROSCI.18-01-00371.1998 PMC6793387

[B36] Chang Z. Ballou E. Jiao W. McKenna K. E. Morrison S. F. McCrimmon D. R. (2013). Systemic leptin produces a long-lasting increase in respiratory motor output in rats. Front. Physiol. 4:16 10.3389/fphys.2013.00016 23408476 PMC3569609

[B37] Chen C. Y. Horowitz J. M. Bonham A. C. (1999). A presynaptic mechanism contributes to depression of autonomic signal transmission in NTS. Am. J. Physiol. 277, H1350–H1360 10516169 10.1152/ajpheart.1999.277.4.H1350

[B38] Ciriello J. Moreau J. M. (2012). Leptin signaling in the nucleus of the solitary tract alters the cardiovascular responses to activation of the chemoreceptor reflex. Am. J. Physiol. Regul. Integr. Comp. Physiol. 303, R727–R736 10.1152/ajpregu.00068.2012 22914750

[B39] Colombari E. Menani J. V. Talman W. T. (1996). Commissural NTS contributes to pressor responses to glutamate injected into the medial NTS of awake rats. Am. J. Physiol. 270, R1220–R1225 8764286 10.1152/ajpregu.1996.270.6.R1220

[B40] Considine R. V. Sinha M. K. Heiman M. L. Kriauciunas A. Stephens T. W. Nyce M. R. (1996). Serum immunoreactive-leptin concentrations in normal-weight and obese humans. N. Engl. J. Med. 334, 292–295 10.1056/NEJM199602013340503 8532024

[B41] Costa-Silva J. H. Zoccal D. B. Machado B. H. (2010). Glutamatergic antagonism in the NTS decreases post-inspiratory drive and changes phrenic and sympathetic coupling during chemoreflex activation. J. Neurophysiol. 103, 2095–2106 10.1152/jn.00802.2009 20164386

[B42] Costa-Silva J. H. Zoccal D. B. Machado B. H. (2012). Chronic intermittent hypoxia alters glutamatergic control of sympathetic and respiratory activities in the commissural NTS of rats. Am. J. Physiol. Regul. Integr. Comp. Physiol. 302, R785–R793 10.1152/ajpregu.00363.2011 22204959

[B43] Cruz J. C. Bonagamba L. G. Stern J. E. Machado B. H. (2010). Fos expression in the NTS in response to peripheral chemoreflex activation in awake rats. Auton. Neurosci. 152, 27–34 10.1016/j.autneu.2009.08.016 19783484

[B44] Czyzyk-Krzeska M. F. Trzebski A. (1990). Respiratory-related discharge pattern of sympathetic nerve activity in the spontaneously hypertensive rat. J. Physiol. 426, 355–368 2231403 10.1113/jphysiol.1990.sp018142 PMC1189892

[B45] Dampney R. A. (1994). Functional organization of central pathways regulating the cardiovascular system. Physiol. Rev. 74, 323–364 8171117 10.1152/physrev.1994.74.2.323

[B46] De Castro D. Lipski J. Kanjhan R. (1994). Electrophysiological study of dorsal respiratory neurons in the medulla oblongata of the rat. Brain Res. 639, 49–56 10.1016/0006-8993(94)91763-9 8180838

[B47] Dempsey J. A. Veasey S. C. Morgan B. J. O'Donnell C. P. (2010). Pathophysiology of sleep apnea. Physiol. Rev. 90, 47–112 10.1152/physrev.00043.2008 20086074 PMC3970937

[B48] De Paula P. M. Tolstykh G. Mifflin S. (2007). Chronic intermittent hypoxia alters NMDA and AMPA-evoked currents in NTS neurons receiving carotid body chemoreceptor inputs. Am. J. Physiol. Regul. Integr. Comp. Physiol. 292, R2259–R2265 10.1152/ajpregu.00760.2006 17332161

[B49] Dick T. E. Hsieh Y. H. Morrison S. Coles S. K. Prabhakar N. (2004). Entrainment pattern between sympathetic and phrenic nerve activities in the Sprague-Dawley rat: hypoxia-evoked sympathetic activity during expiration. Am. J. Physiol. Regul. Integr. Comp. Physiol. 286, R1121–R1128 10.1152/ajpregu.00485.2003 15001434

[B50] Dobbins E. G. Feldman J. L. (1994). Brainstem network controlling descending drive to phrenic motoneurons in rat. J. Comp. Neurol. 347, 64–86 10.1002/cne.903470106 7798382

[B51] Doyle M. W. Bailey T. W. Jin Y. H. Appleyard S. M. Low M. J. Andresen M. C. (2004). Strategies for cellular identification in nucleus tractus solitarius slices. J. Neurosci. Methods 137, 37–48 10.1016/j.jneumeth.2004.02.007 15196825

[B52] Elias C. F. Kelly J. F. Lee C. E. Ahima R. S. Drucker D. J. Saper C. B. (2000). Chemical characterization of leptin-activated neurons in the rat brain. J. Comp. Neurol. 423, 261–281 10.1002/1096-9861(20000724)423:2%3C261::AID-CNE6%3E3.3.CO;2-Y 10867658

[B53] Elmquist J. K. Bjorbaek C. Ahima R. S. Flier J. S. Saper C. B. (1998). Distributions of leptin receptor mRNA isoforms in the rat brain. J. Comp. Neurol. 395, 535–547 10.1002/(SICI)1096-9861(19980615)395:4<535::AID-CNE9>3.0.CO;2-2 9619505

[B54] Esler M. (2012). The sympathetic nervous system through the ages: from Thomas Willis to resistant hypertension. Exp. Physiol. 96, 611–622 10.1113/expphysiol.2010.052332 21551268

[B55] Ezure K. Tanaka I. Saito Y. Otake K. (2002). Axonal projections of pulmonary slowly adapting receptor relay neurons in the rat. J. Comp. Neurol. 446, 81–94 10.1002/cne.10185 11920722

[B56] Fatouleh R. Macefield V. G. (2011). Respiratory modulation of muscle sympathetic nerve activity is not increased in essential hypertension or chronic obstructive pulmonary disease. J. Physiol. 589, 4997–5006 10.1113/jphysiol.2011.210534 21844003 PMC3224888

[B57] Fletcher E. C. Lesske J. Behm R. Miller C. C. 3rd Stauss H. Unger T. (1992). Carotid chemoreceptors, systemic blood pressure, and chronic episodic hypoxia mimicking sleep apnea. J. Appl. Physiol. 72, 1978–1984 1601808 10.1152/jappl.1992.72.5.1978

[B58] Furuya W. I. Bassi M. Menani J. V. Colombari E. Zoccal D. B. Colombari D. S. (2014). Differential modulation of sympathetic and respiratory activities by cholinergic mechanisms in the nucleus of the solitary tract in rats. Exp. Physiol. 99, 743–758 10.1113/expphysiol.2013.076794 24610833

[B59] Galic S. Oakhill J. S. Steinberg G. R. (2010). Adipose tissue as an endocrine organ. Mol. Cell. Endocrinol. 316, 129–139 10.1016/j.mce.2009.08.018 19723556

[B60] Gerber U. Polosa C. (1978). Effects of pulmonary stretch receptor afferent stimulation on sympathetic preganglionic neuron firing. Can. J. Physiol. Pharmacol. 56, 191–198 10.1139/y78-027 638870

[B61] Giardino N. D. Glenny R. W. Borson S. Chan L. (2003). Respiratory sinus arrhythmia is associated with efficiency of pulmonary gas exchange in healthy humans. Am. J. Physiol. Heart Circ. Physiol. 284, H1585–H1591 10.1152/ajpheart.00893.2002 12543637

[B62] Gilbey M. P. (2007). Sympathetic rhythms and nervous integration. Clin. Exp. Pharmacol. Physiol. 34, 356–361 10.1111/j.1440-1681.2007.04587.x 17324150

[B63] Gordon F. J. Sved A. F. (2002). Neurotransmitters in central cardiovascular regulation: glutamate and GABA. Clin. Exp. Pharmacol. Physiol. 29, 522–524 10.1046/j.1440-1681.2002.03666.x 12010202

[B64] Gourine A. V. Dale N. Korsak A. Llaudet E. Tian F. Huckstepp R. (2008). Release of ATP and glutamate in the nucleus tractus solitarii mediate pulmonary stretch receptor (Breuer-Hering) reflex pathway. J. Physiol. 586, 3963–3978 10.1113/jphysiol.2008.154567 18617567 PMC2538935

[B65] Gozal D. Xue Y. D. Simakajornboon N. (1999). Hypoxia induces c-Fos protein expression in NMDA but not AMPA glutamate receptor labeled neurons within the nucleus tractus solitarii of the conscious rat. Neurosci. Lett. 262, 93–96 10.1016/S0304-3940(99)00065-8 10203239

[B66] Grill H. J. (2006). Distributed neural control of energy balance: contributions from hindbrain and hypothalamus. Obesity (Silver Spring) 14 Suppl. 5, 216S–221S 10.1038/oby.2006.312 17021370

[B67] Grill H. J. Hayes M. R. (2009). The nucleus tractus solitarius: a portal for visceral afferent signal processing, energy status assessment and integration of their combined effects on food intake. Int. J. Obes. 33, S11–S15 10.1038/ijo.2009.10 19363500

[B68] Grossman P. Taylor E. W. (2007). Toward understanding respiratory sinus arrhythmia: relations to cardiac vagal tone, evolution and biobehavioral functions. Biol. Psychol. 74, 263–285 10.1016/j.biopsycho.2005.11.014 17081672

[B69] Guertzenstein P. G. Silver A. (1974). Fall in blood pressure produced from discrete regions of the ventral surface of the medulla by glycine and lesions. J. Physiol. 242, 489–503 4455831 10.1113/jphysiol.1974.sp010719 PMC1330679

[B70] Haibara A. S. Colombari E. Chianca D. A. Jr. Bonagamba L. G. Machado B. H. (1995). NMDA receptors in NTS are involved in bradycardic but not in pressor response of chemoreflex. Am. J. Physiol. 269, H1421–H1427 7485576 10.1152/ajpheart.1995.269.4.H1421

[B71] Haibara A. S. Tamashiro E. Olivan M. V. Bonagamba L. G. Machado B. H. (2002). Involvement of the parabrachial nucleus in the pressor response to chemoreflex activation in awake rats. Auton. Neurosci. 101, 60–67 10.1016/S1566-0702(02)00210-2 12462360

[B72] Hall J. E. Da Silva A. A. Do Carmo J. M. Dubinion J. Hamza S. Munusamy S. (2010). Obesity-induced hypertension: role of sympathetic nervous system, leptin, and melanocortins. J. Biol. Chem. 285, 17271–17276 10.1074/jbc.R110.113175 20348094 PMC2878489

[B73] Harsch I. A. Konturek P. C. Koebnick C. Kuehnlein P. P. Fuchs F. S. Pour Schahin S. (2003). Leptin and ghrelin levels in patients with obstructive sleep apnoea: effect of CPAP treatment. Eur. Respir. J. 22, 251–257 10.1183/09031936.03.00010103 12952256

[B74] Haselton J. R. Guyenet P. G. (1989). Central respiratory modulation of medullary sympathoexcitatory neurons in rat. Am. J. Physiol. 256, R739–R750 2923261 10.1152/ajpregu.1989.256.3.R739

[B75] Hayano J. Yasuma F. Okada A. Mukai S. Fujinami T. (1996). Respiratory sinus arrhythmia. A phenomenon improving pulmonary gas exchange and circulatory efficiency. Circulation 94, 842–847 10.1161/01.CIR.94.4.842 8772709

[B76] Hayashi F. Coles S. K. McCrimmon D. R. (1996). Respiratory neurons mediating the Breuer-Hering reflex prolongation of expiration in rat. J. Neurosci. 16, 6526–6536 8815930 10.1523/JNEUROSCI.16-20-06526.1996 PMC6578911

[B77] Hering E. (1868). Die selbststeurung der athmung durch den nervus vagus. Akad. Wiss. Wien (II) 58, 672–677

[B78] Hirooka Y. Sakai K. Kishi T. Ito K. Shimokawa H. Takeshita A. (2003). Enhanced depressor response to endothelial nitric oxide synthase gene transfer into the nucleus tractus solitarii of spontaneously hypertensive rats. Hypertens. Res. 26, 325–331 10.1291/hypres.26.325 12733701

[B79] Inyushkin A. N. Inyushkina E. M. Merkulova N. A. (2009). Respiratory responses to microinjections of leptin into the solitary tract nucleus. Neurosci. Behav. Physiol. 39, 231–240 10.1007/s11055-009-9124-8 19234801

[B80] Inyushkina E. M. Merkulova N. A. Inyushkin A. N. (2010). Mechanisms of the respiratory activity of leptin at the level of the solitary tract nucleus. Neurosci. Behav. Physiol. 40, 707–713 10.1007/s11055-010-9316-2 20635220

[B81] Johnson A. K. Thunhorst R. L. (1997). The neuroendocrinology of thirst and salt appetite: visceral sensory signals and mechanisms of central integration. Front. Neuroendocrinol. 18, 292–353 10.1006/frne.1997.0153 9237080

[B82] Kalia M. Mesulam M. M. (1980). Brain stem projections of sensory and motor components of the vagus complex in the cat: II. Laryngeal, tracheobronchial, pulmonary, cardiac, and gastrointestinal branches. J. Comp. Neurol. 193, 467–508 10.1002/cne.901930211 7440778

[B83] Kershaw E. E. Flier J. S. (2004). Adipose tissue as an endocrine organ. J. Clin. Endocrinol. Metab. 89, 2548–2556 10.1210/jc.2004-0395 15181022

[B84] King T. L. Heesch C. M. Clark C. G. Kline D. D. Hasser E. M. (2012). Hypoxia activates nucleus tractus solitarii neurons projecting to the paraventricular nucleus of the hypothalamus. Am. J. Physiol. Regul. Integr. Comp. Physiol. 302, R1219–R1232 10.1152/ajpregu.00028.2012 22403798 PMC3362152

[B85] Kline D. D. (2008). Plasticity in glutamatergic NTS neurotransmission. Respir. Physiol. Neurobiol. 164, 105–111 10.1016/j.resp.2008.04.013 18524694 PMC2666915

[B86] Kline D. D. Ramirez-Navarro A. Kunze D. L. (2007). Adaptive depression in synaptic transmission in the nucleus of the solitary tract after *in vivo* chronic intermittent hypoxia: evidence for homeostatic plasticity. J. Neurosci. 27, 4663–4673 10.1523/JNEUROSCI.4946-06.2007 17460079 PMC6673010

[B87] Koshiya N. Guyenet P. G. (1994). A5 noradrenergic neurons and the carotid sympathetic chemoreflex. Am. J. Physiol. 267, R519–R526 8067463 10.1152/ajpregu.1994.267.2.R519

[B88] Koshiya N. Guyenet P. G. (1996a). NTS neurons with carotid chemoreceptor inputs arborize in the rostral ventrolateral medulla. Am. J. Physiol. 270, R1273–R1278 8764294 10.1152/ajpregu.1996.270.6.R1273

[B89] Koshiya N. Guyenet P. G. (1996b). Tonic sympathetic chemoreflex after blockade of respiratory rhythmogenesis in the rat. J. Physiol. 491(Pt 3), 859–869 8815217 10.1113/jphysiol.1996.sp021263 PMC1158824

[B90] Kubin L. Alheid G. F. Zuperku E. J. McCrimmon D. R. (2006). Central pathways of pulmonary and lower airway vagal afferents. J. Appl. Physiol. 101, 618–627 10.1152/japplphysiol.00252.2006 16645192 PMC4503231

[B91] Kubo T. Hagiwara Y. Sekiya D. Fukumori R. (1998). Evidence for involvement of the lateral parabrachial nucleus in mediation of cholinergic inputs to neurons in the rostral ventrolateral medulla of the rat. Brain Res. 789, 23–31 10.1016/S0006-8993(97)01452-2 9602037

[B92] Kumada M. Terui N. Kuwaki T. (1990). Arterial baroreceptor reflex: its central and peripheral neural mechanisms. Prog. Neurobiol. 35, 331–361 10.1016/0301-0082(90)90036-G 2263735

[B93] Lahiri S. Roy A. Baby S. M. Hoshi T. Semenza G. L. Prabhakar N. R. (2006). Oxygen sensing in the body. Prog. Biophys. Mol. Biol. 91, 249–286 10.1016/j.pbiomolbio.2005.07.001 16137743

[B94] Lindsey B. G. Arata A. Morris K. F. Hernandez Y. M. Shannon R. (1998). Medullary raphe neurones and baroreceptor modulation of the respiratory motor pattern in the cat. J. Physiol. 512(Pt 3), 863–882 10.1111/j.1469-7793.1998.863bd.x 9769428 PMC2231246

[B95] Loewy A. D. (1990). Central autonomic pathways, in Central Regulation of Autonomic Functions, eds Loewy A. D. Spyer K. M. (New York, NY: Oxford University Press), 88–103

[B96] Machado B. H. (2001). Neurotransmission of the cardiovascular reflexes in the nucleus tractus solitarii of awake rats. Ann. N.Y. Acad. Sci. 940, 179–196 10.1111/j.1749-6632.2001.tb03676.x 11458676

[B97] Machado B. H. Bonagamba L. G. (2005). Antagonism of glutamate receptors in the intermediate and caudal NTS of awake rats produced no changes in the hypertensive response to chemoreflex activation. Auton. Neurosci. 117, 25–32 10.1016/j.autneu.2004.10.004 15620567

[B98] Machado B. H. Castania J. A. Bonagamba L. G. Salgado H. C. (2000). Neurotransmission of autonomic components of aortic baroreceptor afferents in the NTS of awake rats. Am. J. Physiol. Heart Circ. Physiol. 279, H67–H75 10899042 10.1152/ajpheart.2000.279.1.H67

[B99] Malli F. Papaioannou A. I. Gourgoulianis K. I. Daniil Z. (2010). The role of leptin in the respiratory system: an overview. Respir. Res. 11:152 10.1186/1465-9921-11-152 21040518 PMC2988727

[B100] Malpas S. C. (1998). The rhythmicity of sympathetic nerve activity. Prog. Neurobiol. 56, 65–96 10.1016/S0301-0082(98)00030-6 9723131

[B101] Mandel D. A. Schreihofer A. M. (2006). Central respiratory modulation of barosensitive neurones in rat caudal ventrolateral medulla. J. Physiol. 572, 881–896 10.1113/jphysiol.2005.103622 16527859 PMC1780020

[B102] Mandel D. A. Schreihofer A. M. (2009). Modulation of the sympathetic response to acute hypoxia by the caudal ventrolateral medulla in rats. J. Physiol. 587, 461–475 10.1113/jphysiol.2008.161760 19047207 PMC2670056

[B103] Marchenko V. Sapru H. N. (2000). Different patterns of respiratory and cardiovascular responses elicited by chemical stimulation of dorsal medulla in the rat. Brain Res. 857, 99–109 10.1016/S0006-8993(99)02377-X 10700557

[B104] Mark A. L. Agassandian K. Morgan D. A. Liu X. Cassell M. D. Rahmouni K. (2009). Leptin signaling in the nucleus tractus solitarii increases sympathetic nerve activity to the kidney. Hypertension 53, 375–380 10.1161/HYPERTENSIONAHA.108.124255 19103999 PMC2688398

[B105] McAllen R. M. (1987). Central respiratory modulation of subretrofacial bulbospinal neurones in the cat. J. Physiol. 388, 533–545 3116217 10.1113/jphysiol.1987.sp016630 PMC1192564

[B106] McBryde F. D. Abdala A. P. Hendy E. B. Pijacka W. Marvar P. Moraes D. J. (2013). The carotid body as a putative therapeutic target for the treatment of neurogenic hypertension. Nat. Commun. 4:2395 10.1038/ncomms3395 24002774

[B107] McCrimmon D. R. Speck D. F. Feldman J. L. (1987). Role of the ventrolateral region of the nucleus of the tractus solitarius in processing respiratory afferent input from vagus and superior laryngeal nerves. Exp. Brain Res. 67, 449–459 10.1007/BF00247278 3653307

[B108] Mei L. Zhang J. Mifflin S. (2003). Hypertension alters GABA receptor-mediated inhibition of neurons in the nucleus of the solitary tract. Am. J. Physiol. Regul. Integr. Comp. Physiol. 285, R1276–R1286 10.1152/ajpregu.00255.2003 14615399

[B109] Mifflin S. W. (1992). Arterial chemoreceptor input to nucleus tractus solitarius. Am. J. Physiol. 263, R368–R375 1510176 10.1152/ajpregu.1992.263.2.R368

[B110] Mifflin S. W. Felder R. B. (1990). Synaptic mechanisms regulating cardiovascular afferent inputs to solitary tract nucleus. Am. J. Physiol. 259, H653–H661 2204275 10.1152/ajpheart.1990.259.3.H653

[B111] Mifflin S. W. Spyer K. M. Withington-Wray D. J. (1988). Baroreceptor inputs to the nucleus tractus solitarius in the cat: modulation by the hypothalamus. J. Physiol. 399, 369–387 3404464 10.1113/jphysiol.1988.sp017086 PMC1191670

[B112] Miyawaki T. Pilowsky P. Sun Q. J. Minson J. Suzuki S. Arnolda L. (1995). Central inspiration increases barosensitivity of neurons in rat rostral ventrolateral medulla. Am. J. Physiol. 268, R909–R918 7733401 10.1152/ajpregu.1995.268.4.R909

[B113] Molkov Y. I. Bacak B. J. Dick T. E. Rybak I. A. (2013). Control of breathing by interacting pontine and pulmonary feedback loops. Front. Neural Circuits 7:16 10.3389/fncir.2013.00016 23408512 PMC3570896

[B114] Molkov Y. I. Zoccal D. B. Moraes D. J. Paton J. F. Machado B. H. Rybak I. A. (2011). Intermittent hypoxia-induced sensitization of central chemoreceptors contributes to sympathetic nerve activity during late expiration in rats. J. Neurophysiol. 105, 3080–3091 10.1152/jn.00070.2011 21471394 PMC3118734

[B115] Moraes D. J. Bonagamba L. G. Zoccal D. B. Machado B. H. (2011). Modulation of respiratory responses to chemoreflex activation by L-glutamate and ATP in the rostral ventrolateral medulla of awake rats. Am. J. Physiol. Regul. Integr. Comp. Physiol. 300, R1476–R1486 10.1152/ajpregu.00825.2010 21411762

[B116] Moraes D. J. Da Silva M. P. Bonagamba L. G. Mecawi A. S. Zoccal D. B. Antunes-Rodrigues J. (2013). Electrophysiological properties of rostral ventrolateral medulla presympathetic neurons modulated by the respiratory network in rats. J. Neurosci. 33, 19223–19237 10.1523/JNEUROSCI.3041-13.2013 24305818 PMC6618786

[B117] Moraes D. J. Dias M. B. Cavalcanti-Kwiatkoski R. Machado B. H. Zoccal D. B. (2012a). Contribution of retrotrapezoid/parafacial respiratory region to the expiratory-sympathetic coupling in response to peripheral chemoreflex in rats. J. Neurophysiol. 108, 882–890 10.1152/jn.00193.2012 22592303

[B118] Moraes D. J. Machado B. H. Paton J. F. (2014). Specific respiratory neuron types have increased excitability that drive presympathetic neurones in neurogenic hypertension. Hypertension 63, 1309–1318 10.1161/HYPERTENSIONAHA.113.02283 24688126

[B119] Moraes D. J. Zoccal D. B. Machado B. H. (2012b). Medullary respiratory network drives sympathetic overactivity and hypertension in rats submitted to chronic intermittent hypoxia. Hypertension 60, 1374–1380 10.1161/HYPERTENSIONAHA.111.189332 23108658

[B120] Moraes D. J. Zoccal D. B. Machado B. H. (2012c). Sympathoexcitation during chemoreflex active expiration is mediated by L-glutamate in the RVLM/Botzinger complex of rats. J. Neurophysiol. 108, 610–623 10.1152/jn.00057.2012 22539823

[B121] Narkiewicz K. Van De Borne P. J. H. Montano N. Dyken M. E. Phillips B. G. Somers V. K. (1998). Contribution of tonic chemoreflex activation to sympathetic activity and blood pressure in patients with obstructive sleep apnea. Circulation 97, 943–945 10.1161/01.CIR.97.10.943 9529260

[B122] Niewinski P. Janczak D. Rucinski A. Jazwiec P. Sobotka P. A. Engelman Z. J. (2013). Carotid body removal for treatment of chronic systolic heart failure. Int. J. Cardiol. 168, 2506–2509 10.1016/j.ijcard.2013.03.011 23541331

[B123] Olivan M. V. Bonagamba L. G. Machado B. H. (2001). Involvement of the paraventricular nucleus of the hypothalamus in the pressor response to chemoreflex activation in awake rats. Brain Res. 895, 167–172 10.1016/S0006-8993(01)02067-4 11259774

[B124] Patel S. R. Palmer L. J. Larkin E. K. Jenny N. S. White D. P. Redline S. (2004). Relationship between obstructive sleep apnea and diurnal leptin rhythms. Sleep 27, 235–239 15124716 10.1093/sleep/27.2.235

[B125] Peña F. Ramirez J.-M. (2005). Hypoxia-induced changes in neuronal network properties. Mol. Neurobiol. 32, 251–283 10.1385/MN:32:3:251 16385141

[B126] Peng Y.-J. Overholt J. L. Kline D. Kumar G. K. Prabhakar N. R. (2003). Induction of sensory long-term facilitation in the carotid body by intermittent hypoxia: implications for recurrent apneas. Proc. Natl. Acad. Sci. U.S.A. 100, 10073–10078 10.1073/pnas.1734109100 12907705 PMC187770

[B127] Peters J. H. McDougall S. J. Kellett D. O. Jordan D. Llewellyn-Smith I. J. Andresen M. C. (2008). Oxytocin enhances cranial visceral afferent synaptic transmission to the solitary tract nucleus. J. Neurosci. 28, 11731–11740 10.1523/JNEUROSCI.3419-08.2008 18987209 PMC2585803

[B128] Queiroz E. A. Okada M. N. Fumega U. Fontes M. A. Moraes M. F. Haibara A. S. (2011). Excitatory amino acid receptors in the dorsomedial hypothalamus are involved in the cardiovascular and behavioural chemoreflex responses. Exp. Physiol. 96, 73–84 10.1113/expphysiol.2010.054080 20889605

[B129] Rahmouni K. Morgan D. A. Morgan G. M. Mark A. L. Haynes W. G. (2005). Role of selective leptin resistance in diet-induced obesity hypertension. Diabetes 54, 2012–2018 10.2337/diabetes.54.7.2012 15983201

[B130] Reddy M. K. Patel K. P. Schultz H. D. (2005). Differential role of the paraventricular nucleus of the hypothalamus in modulating the sympathoexcitatory component of peripheral and central chemoreflexes. Am. J. Physiol. Regul. Integr. Comp. Physiol. 289, R789–R797 10.1152/ajpregu.00222.2005 15919733

[B131] Reeves S. R. Gozal E. Guo S. Z. Sachleben L. R. Jr. Brittian K. R. Lipton A. J. (2003). Effect of long-term intermittent and sustained hypoxia on hypoxic ventilatory and metabolic responses in the adult rat. J. Appl. Physiol. 95, 1767–1774 10.1152/japplphysiol.00759.2002 14555663

[B132] Reis D. J. Granata A. R. Perrone M. H. Talman W. T. (1981). Evidence that glutamic acid is the neurotransmitter of baroreceptor afferent terminating in the nucleus tractus solitarius (NTS). J. Auton. Nerv. Syst. 3, 321–334 10.1016/0165-1838(81)90073-4 6115875

[B133] Rey S. Del Rio R. Alcayaga J. Iturriaga R. (2004). Chronic intermittent hypoxia enhances cat chemosensory and ventilatory responses to hypoxia. J. Physiol. 560, 577–586 10.1113/jphysiol.2004.072033 15319419 PMC1665248

[B134] Richter D. W. Seller H. (1975). Baroreceptor effects on medullary respiratory neurones of the cat. Brain Res. 86, 168–171 10.1016/0006-8993(75)90651-4 163666

[B135] Ross C. A. Ruggiero D. A. Park D. H. Joh T. H. Sved A. F. Fernandez-Pardal J. (1984). Tonic vasomotor control by the rostral ventrolateral medulla: effect of electrical or chemical stimulation of the area containing C1 adrenaline neurons on arterial pressure, heart rate, and plasma catecholamines and vasopressin. J. Neurosci. 4, 474–494 6699683 10.1523/JNEUROSCI.04-02-00474.1984 PMC6564896

[B136] Ruggiero D. A. Giuliano R. Anwar M. Stornetta R. Reis D. J. (1990). Anatomical substrates of cholinergic-autonomic regulation in the rat. J. Comp. Neurol. 292, 1–53 10.1002/cne.902920102 2312784

[B137] Sapru H. N. (1996). Carotid chemoreflex. Neural pathways and transmitters. Adv. Exp. Med. Biol. 410, 357–364 10.1007/978-1-4615-5891-0_55 9030325

[B138] Sato M. A. Colombari E. Morrison S. F. (2002). Inhibition of neurons in commissural nucleus of solitary tract reduces sympathetic nerve activity in SHR. Am. J. Physiol. Heart Circ. Physiol. 282, H1679–H1684 10.1152/ajpheart.00619.2001 11959631

[B139] Sato M. A. Menani J. V. Ubríaco-Lopes O. Colombari E. (2001). Lesions of the commissural nucleus of the solitary tract reduce arterial pressure in spontaneously hypertensive rats. Hypertension 38, 560–564 10.1161/01.HYP.38.3.560 11566931

[B140] Schafer H. Pauleit D. Sudhop T. Gouni-Berthold I. Ewig S. Berthold H. K. (2002). Body fat distribution, serum leptin, and cardiovascular risk factors in men with obstructive sleep apnea. Chest 122, 829–839 10.1378/chest.122.3.829 12226021

[B141] Schreihofer A. M. Guyenet P. G. (2003). Baro-activated neurons with pulse-modulated activity in the rat caudal ventrolateral medulla express GAD67 mRNA. J. Neurophysiol. 89, 1265–1277 10.1152/jn.00737.2002 12612005

[B142] Sellden H. Delle M. Sjovall H. Ricksten S. E. (1987). Reflex changes in sympathetic nerve activity during mechanical ventilation with PEEP in sino-aortic denervated rats. Acta Physiol. Scand. 130, 15–24 10.1111/j.1748-1716.1987.tb08106.x 3296660

[B143] Shan Z. Zubcevic J. Shi P. Jun J. Y. Dong Y. Murca T. M. (2013). Chronic knockdown of the nucleus of the solitary tract AT1 receptors increases blood inflammatory-endothelial progenitor cell ratio and exacerbates hypertension in the spontaneously hypertensive rat. Hypertension 61, 1328–1333 10.1161/HYPERTENSIONAHA.111.00156 23547238 PMC4128231

[B144] Shigetomi E. Kato F. (2004). Action potential-independent release of glutamate by Ca2+ entry through presynaptic P2X receptors elicits postsynaptic firing in the brainstem autonomic network. J. Neurosci. 24, 3125–3135 10.1523/JNEUROSCI.0090-04.2004 15044552 PMC6729830

[B145] Shihara M. Hori N. Hirooka Y. Eshima K. Akaike N. Takeshita A. (1999). Cholinergic systems in the nucleus of the solitary tract of rats. Am. J. Physiol. 276, R1141–R1148 10198396 10.1152/ajpregu.1999.276.4.R1141

[B146] Simms A. E. Paton J. F. Pickering A. E. Allen A. M. (2009). Amplified respiratory-sympathetic coupling in the spontaneously hypertensive rat: does it contribute to hypertension? J. Physiol. 587, 597–610 10.1113/jphysiol.2008.165902 19064613 PMC2670083

[B147] Smith J. C. Abdala A. P. Koizumi H. Rybak I. A. Paton J. F. (2007). Spatial and functional architecture of the mammalian brain stem respiratory network: a hierarchy of three oscillatory mechanisms. J. Neurophysiol. 98, 3370–3387 10.1152/jn.00985.2007 17913982 PMC2225347

[B148] Smith J. C. Ellenberger H. H. Ballanyi K. Richter D. W. Feldman J. L. (1991). Pre-Botzinger complex: a brainstem region that may generate respiratory rhythm in mammals. Science 254, 726–729 10.1126/science.1683005 1683005 PMC3209964

[B149] Somers V. K. (1995). Sympathetic neural mechanisms in obstructive sleep apnea. J. Clin. Investig. 96, 1897–1904 10.1172/JCI118235 7560081 PMC185826

[B150] Song G. Xu H. Wang H. Macdonald S. M. Poon C. S. (2011). Hypoxia-excited neurons in NTS send axonal projections to Kolliker-Fuse/parabrachial complex in dorsolateral pons. Neuroscience 175, 145–153 10.1016/j.neuroscience.2010.11.065 21130843 PMC3035171

[B151] Spary E. J. Maqbool A. Saha S. Batten T. F. (2008). Increased GABA B receptor subtype expression in the nucleus of the solitary tract of the spontaneously hypertensive rat. J. Mol. Neurosci. 35, 211–224 10.1007/s12031-008-9055-9 18338268

[B152] Staessen J. A. Wang J. Bianchi G. Birkenhager W. H. (2003). Essential hypertension. Lancet 361, 1629–1641 10.1016/S0140-6736(03)13302-8 12747893

[B153] Sun Q. J. Minson J. Llewellyn-Smith I. J. Arnolda L. Chalmers J. Pilowsky P. (1997). Botzinger neurons project towards bulbospinal neurons in the rostral ventrolateral medulla of the rat. J. Comp. Neurol. 388, 23–31 10.1002/(SICI)1096-9861(19971110)388:1<23::AID-CNE2>3.0.CO;2-Q[pii] 9364236

[B154] Takakura A. C. Moreira T. S. Colombari E. West G. H. Stornetta R. L. Guyenet P. G. (2006). Peripheral chemoreceptor inputs to retrotrapezoid nucleus (RTN) CO2-sensitive neurons in rats. J. Physiol. 572, 503–523 10.1113/jphysiol.2005.103788 16455687 PMC1779666

[B155] Taxini C. L. Takakura A. C. Gargaglioni L. H. Moreira T. S. (2011). Control of the central chemoreflex by A5 noradrenergic neurons in rats. Neuroscience 199, 177–186 10.1016/j.neuroscience.2011.09.068 22015927

[B156] Teppema L. J. Veening J. G. Kranenburg A. Dahan A. Berkenbosch A. Olievier C. (1997). Expression of c-fos in the rat brainstem after exposure to hypoxia and to normoxic and hyperoxic hypercapnia. J. Comp. Neurol. 388, 169–190 10.1002/(SICI)1096-9861(19971117)388:2<169::AID-CNE1>3.0.CO;2-# 9368836

[B157] Toney G. M. Pedrino G. R. Fink G. D. Osborn J. W. (2010). Does enhanced respiratory-sympathetic coupling contribute to peripheral neural mechanisms of angiotensin II-salt hypertension? Exp. Physiol. 95, 587–594 10.1113/expphysiol.2009.047399 20228120 PMC2978666

[B158] Van Giersbergen P. L. Palkovits M. De Jong W. (1992). Involvement of neurotransmitters in the nucleus tractus solitarii in cardiovascular regulation. Physiol. Rev. 72, 789–824 1352638 10.1152/physrev.1992.72.3.789

[B159] Waki H. Gouraud S. S. Maeda M. Paton J. F. (2008). Specific inflammatory condition in nucleus tractus solitarii of the SHR: novel insight for neurogenic hypertension? Auton. Neurosci. 142, 25–31 10.1016/j.autneu.2008.07.003 18722165

[B160] Yu J. Roberts A. M. Joshua I. G. (1990). Lung inflation evokes reflex dilation of microvessels in rat skeletal muscle. Am. J. Physiol. 258, H939–H945 2331017 10.1152/ajpheart.1990.258.4.H939

[B161] Zhang W. Carreno F. R. Cunningham J. T. Mifflin S. W. (2008). Chronic sustained and intermittent hypoxia reduce function of ATP-sensitive potassium channels in nucleus of the solitary tract. Am. J. Physiol. Regul. Integr. Comp. Physiol. 295, R1555–R1562 10.1152/ajpregu.90390.2008 18784334 PMC2584857

[B162] Zhang W. Mifflin S. (2010). Chronic hypertension enhances presynaptic inhibition by baclofen in the nucleus of the solitary tract. Hypertension 55, 481–486 10.1161/HYPERTENSIONAHA.109.145151 20038748 PMC2836185

[B163] Zhang W. Mifflin S. W. (1993). Excitatory amino acid receptors within NTS mediate arterial chemoreceptor reflexes in rats. Am. J. Physiol. 265, H770–H773 8368379 10.1152/ajpheart.1993.265.2.H770

[B164] Zhou S. Y. Gilbey M. P. (1992). Respiratory-related activity of lower thoracic and upper lumbar sympathetic preganglionic neurones in the rat. J. Physiol. 451, 631–642 1403828 10.1113/jphysiol.1992.sp019182 PMC1176179

[B165] Zhou Z. Champagnat J. Poon C. S. (1997). Phasic and long-term depression in brainstem nucleus tractus solitarius neurons: differing roles of AMPA receptor desensitization. J. Neurosci. 17, 5349–5356 9204919 10.1523/JNEUROSCI.17-14-05349.1997 PMC6793832

[B166] Zoccal D. B. Bonagamba L. G. Oliveira F. R. Antunes-Rodrigues J. Machado B. H. (2007). Increased sympathetic activity in rats submitted to chronic intermittent hypoxia. Exp. Physiol. 92, 79–85 10.1113/expphysiol.2006.035501 17085676

[B167] Zoccal D. B. Bonagamba L. G. Paton J. F. Machado B. H. (2009). Sympathetic-mediated hypertension of awake juvenile rats submitted to chronic intermittent hypoxia is not linked to baroreflex dysfunction. Exp. Physiol. 94, 972–983 10.1113/expphysiol.2009.048306 19578126

[B168] Zoccal D. B. Simms A. E. Bonagamba L. G. Braga V. A. Pickering A. E. Paton J. F. (2008). Increased sympathetic outflow in juvenile rats submitted to chronic intermittent hypoxia correlates with enhanced expiratory activity. J. Physiol. 586, 3253–3265 10.1113/jphysiol.2008.154187 18450774 PMC2538770

